# Estimation of the global number of e-cigarette users in 2020

**DOI:** 10.1186/s12954-021-00556-7

**Published:** 2021-10-23

**Authors:** Tomasz Jerzyński, Gerry V. Stimson, Harry Shapiro, Grzegorz Król

**Affiliations:** 1 Knowledge-Action-Change, 8 Northumberland Avenue, London, WC2N 5BY UK; 2 University of Warsaw, Warsaw, Poland; 3 Imperial College London, London, UK

**Keywords:** E-cigarettes, Nicotine, Vaping products, Tobacco harm reduction, Estimation methods

## Abstract

**Background:**

The combustion of tobacco is the main cause of tobacco-related morbidity and mortality. E-cigarettes are potentially disruptive innovations with considerable potential for population health. A key question is whether e-cigarettes are replacing tobacco cigarettes, which requires mapping their prevalence. Collecting information on nicotine use is difficult for many countries due to cost. The objective of this study was to derive a global estimate of e-cigarette use (vaping).

**Methods:**

Since 2018 we have collected information on the prevalence of e-cigarette use. To estimate the prevalence of vaping in countries lacking information, we used the method of assumed similarity between countries in the same region and economic condition. Based on surveys, we calculated the average prevalence of vaping for each WHO region, World Bank income classification group, and the legal status of e-cigarettes in each country. For each of these groups the average prevalence of vaping was calculated. These values were used as substitutes for the prevalence figures in the countries with absent data. The number of vapers was calculated by taking as the denominator the adult population.

**Results:**

Survey data on e-cigarette users were available for 49 countries covering 2.8 b of the adult population in 2018 and unavailable for 2.9 b. Information on vaping was lacking for half of the world's population. We estimated a total of 58.1 m vapers worldwide in 2018. By reference to market growth the data were adjusted to arrive at estimates for 2020. Results were fitted to revenue data at the 2018. For the year 2020, the projection is for 68 m vapers globally.

**Conclusions:**

Many global epidemiological studies use the method of assumed similarity between countries with shared characteristics in order to estimate missing data. The methodological limitations are likely to overestimate the global number of vapers. Our estimate of 68 m vapers indicates considerable uptake given that: e-cigarettes have been available on most markets for only a decade; there is either no support, or there is opposition to vaping in many countries; and countries which regulate e-cigarettes have controls over advertising and promotion. However, given the global scale of tobacco smoking (at 1.1 billion people), progress in adoption of alternative products is slow. Those using e-cigarettes are still a small fraction of those who smoke.

**Supplementary Information:**

The online version contains supplementary material available at 10.1186/s12954-021-00556-7.

## Introduction

Nicotine is one of the world’s most popular drugs, alongside alcohol and caffeine. Most users, around 68%, consume nicotine by smoking tobacco cigarettes. Safer nicotine products (SNP) including e-cigarettes provide a non-combustible means for consuming nicotine. A key question from a public health perspective is the extent of use of these products, which have been available in many markets since 2007. The World Health Organization (WHO) estimates that there are 1.1 billion smokers globally. This figure has remained unchanged since 2000 and is equivalent to one in five of the global adult population [1]. Declines in the prevalence of smoking in some countries and population groups have been offset by increases elsewhere and by population increases.. 80% of smokers live in low and middle income countries [1, 2]. The health effects of smoking tobacco are well known with an estimate of 7 million premature deaths from smoking related disease each year [3], which is three times more than the number who die from malaria, HIV and tuberculosis combined.

There has been rapid uptake of e-cigarettes in some countries in Europe and in North America. In the UK the percentage of the adult population using e-cigarettes rose from 1.7% in 2012 to 7.1% in 2019, and in the same time smoking rates dropped from 19.6 to 14.7% [4]. The prevalence of vaping in European Union countries varies between less than 0.2% and more than 7% [4–6]. The uptake of e-cigarettes, and indeed of other non-combustible SNP such as heated tobaccos and Swedish snus, is an unusual public health phenomenon because it has been driven by consumers and products rather than by formal public health agencies.

Mapping the use of tobacco and nicotine products is patchy. The WHO promotes the MPOWER tobacco control programme, the first letter of which refers to Monitoring [7]. One of the key points of monitoring is the collection of up-to-date information on the use of tobacco and related products. This is an extremely difficult task for many countries due to the cost involved. Given this dearth of information on tobacco use, the WHO uses a variety of epidemiological techniques to develop estimates that are based on partial and sometimes unrepresentative data.

The situation regarding population measurement of e-cigarettes is even more challenging given the relatively recent arrival and uptake of these products. Ideally information on vaping should come from nationally representative surveys, or extrapolation from geographically limited surveys. Some of the information on vaping comes from convenience samples including internet surveys. The other main source of information is from market research studies. However, the interpretation of these is difficult in population terms, because they focus on units sold or market value, rather than on the number of users. These studies also mainly derive from the store data, and hence miss internet sales and small shops. There is an uncertain relationship between market values and number of users, which depends, amongst other things, on frequency and quantity of use. Most market research is commissioned by manufacturers and is not in the public domain.

E-cigarettes are potentially disruptive innovations which in turn have considerable potential in tobacco harm reduction. A key question is the rate at which e-cigarettes and other SNP are replacing tobacco cigarettes. The objective of this study was to derive a global estimate of the number of people using e-cigarettes.

## Methods

Since 2018 we have been collecting information on the prevalence of vaping as part of the Global State of Tobacco Harm Reduction project (GSTHR). This project maps the global, regional and national availability and use of SNP, the regulatory responses to these products, and the public health potential of tobacco harm reduction. Information is collected from the literature and a network of national and regional correspondents on smoking prevalence and mortality, SNP and nicotine replacement therapy (NRT), use and regulations and controls across 201 countries (262 including territories and significant within-country regions). The database has been designed to compile country-based information over time and product categories. Country level data are available on the GSTHR website (https://gsthr.org). Data on e-cigarette use are reported where available from nationally representative surveys of the general adult population.

Inclusion criteria for the studies used in our analysis were that they were recent (published between 2012 and 2020) general population surveys and included information on daily or current use of e-cigarettes. We found surveys on e-cigarette use from 49 countries (see “Results” section).

The question arises as to how to derive a global estimate of e-cigarette use. The problem of missing data is quite common in the social sciences and epidemiology. Data loss begins at the survey implementation stage. The selected sample is never fully complete. Certain groups of people are always unavailable. Researchers try to avoid statistical error caused by missing data and the most commonly used of these is post-stratification weighting [8–11].

For characteristics aggregated at the country level—as is the case for smoking or prevalence of e-cigarette use—we have almost the same situation as for survey incompleteness. General characteristics can be used to estimate missing information. Information on the prevalence of e-cigarette use comes from surveys that have not been widely conducted in all countries. Moreover, surveys use different definitions and measurement of e-cigarette use, and in some there is lack of clarity regarding which substances are being used. We focused only on rates of current use of e-cigarettes, which is commonly defined as using at least once in the last 30 days, so that includes daily use.

### Estimation methods

Given that there is information for only 49 countries, the question arises as to how to estimate the prevalence of vaping in countries for which information is unavailable. We have used an accepted epidemiological method of estimating country data by assumed similarity between countries in the same region and economic condition for which data points are available. This methodology is commonly used for estimating health status in the absence of national surveys. Aceijas [12] showed one of the variants of this method in her study about relationship between drug injecting and HIV infections. Dawood [13] used it to estimate the mortality associated with pandemic influenza in 2009. And Verity [14] applied a statistical model based on the same assumptions to estimate the prevalence of coronavirus infection. The WHO and other United Nations organisations publish global and national estimates from multiple agencies on a wide range of indicators, particularly mortality levels and trends [15]. A good example is the process used to impute incidence at the country level in the Global Burden of Disease study, which uses three different statistical models to produce reliable estimates [16].

The method is similar to other methods used for statistical inference and analysis with incomplete data. The main mathematical tool used for estimation is a method that can be called average similarity. It assumes that if certain characteristics in a given group of countries are similar, then these countries will also be similar in other respects. This allows us to impute unknown values of the characteristics with the average values of those characteristics in the countries where they are known [17]. This is a major assumption and has obvious limitations.

The main characteristics that can be used are general characteristics such as geographic regions and economic groups. However, health-related behaviours such as nicotine use are related to a wide range of social phenomena, for example religious customs and rituals, historical events, and cultural, economic, and political conditions. A well-conducted operationalization of several parameters that could cover the above spheres would be a great improvement of the estimation procedure. These parameters are rarely available.

**Fig. 1:**
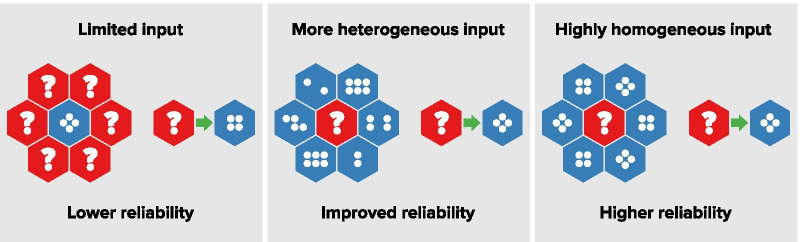
Schematic representation of input data and quality of estimates

The quality of such estimation depends on the ratio of known and unknown fractions (see “Survey population coverage” section). The higher the level of input data and the more homogeneous they are, the better the result. Figure 1 illustrates the relationship between amount and internal differentiation of input data and quality of estimates.

To achieve better control over the quality and reliability of the process, a synthetic goodness-of-fit characteristic can be calculated. It can be based on the standard deviation and the number of cases in the aggregated groups. This is analogous to a simplified confidence interval.

A further issue regards the availability and relevance of data on country characteristics from which similarity between countries may be inferred. We used the WHO region, the World Bank income classification and the legal status of e-cigarettes. Our database (https://gsthr.org) lists countries which ban the sale of these products.

The second methodological problem is that surveys are conducted in different years and are often not repeated. Most of the available data we found allow for the calculation of an estimate for 2018 (32 studies are from 2017 to 2018, 14 are earlier and 3 are later). Therefore, the data need to be adjusted to arrive at estimates for 2020. This can be done by reference to data on market growth. Based on publicly available data on e-cigarette market value reported by Statista [18], we can track changes in global revenues in the e-cigarette market. The relationship between market value and vaping prevalence is unclear, as market values are affected by price and consumption patterns. It would be good for the quality of the projection to know the efficiency of the relationship between market data and the number of vapers in the population. It can easily be calculated where both the local market revenue and the prevalence of vapers are known. Investigating the relationship between market revenues and the prevalence of vaping for countries where both data are available enables forward projection of the global number of vapers in 2020.

## Results

### Vaping surveys—data availability

**Fig. 2:**
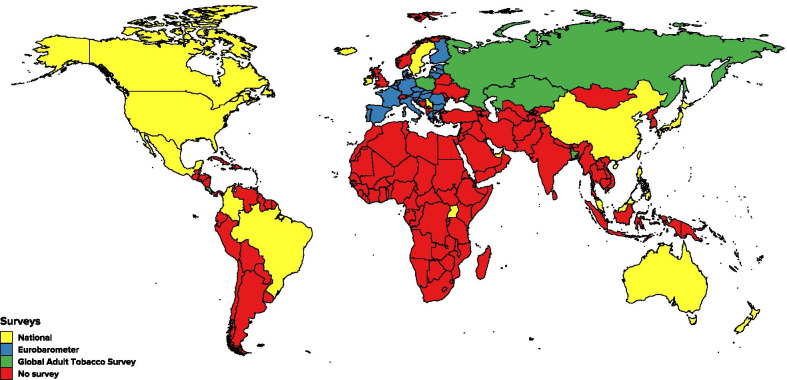
Surveys on vaping

Information on the prevalence of vaping from nationally representative surveys was available for 49 countries (see Fig. 2 and Additional file 1). There are two publicly available international research programmes and eight national programmes on tobacco use and vaping. The international research from which we obtained data comprises: The Global Adult Tobacco Survey (GATS) [19], last conducted in 2017, providing data on six countries (there are 25 of them in the study, but only six were used due to outdated surveys or not covering the subject of e-cigarettes); and the Special Eurobarometer on *Attitudes of Europeans towards tobacco and electronic cigarettes* [5], last conducted in 2017, gathering data from 28 countries.^1^ The International Tobacco Control surveys [20] include 29 countries for which data on e-cigarette use is available in six countries but we did not use it due to methodological issues including the use of non-probabilistic samples.

National surveys on tobacco use and vaping (or at least having some information on this topic) include for example: National Drug Strategy Household Survey 2019 (Australia), Healthy Ireland Survey 2017, Malaysia 2016 National E-Cigarette Survey, Encuesta Nacional de Consumo de Drogas, Alcohol y Tabaco (ENCODAT 2016–2017, Mexico), Action on Smoking and Health survey (Great Britain 2018), the Office for National Statistics (UK 2019), and the National Adult Tobacco Survey (USA, up to 2018). In addition, we obtained information from general surveys for seven other countries.

### Survey population coverage

To be more relevant, it is useful to look at the availability of data from a world population perspective. According to the United Nations, the adult (15 years old or above) population of the countries for which vaping survey data were available was nearly 2.8 billion in 2018. The adult population of the remaining countries was over 2.9 billion. Thus, information on vaping was lacking for just over half of the world's adult population (see Table 1).

**Table 1:** Adult population (15 years old or above) in surveyed and not surveyed countries in 2018

	Surveyed population		Not surveyed population	
	Count	Percent	Count	Percent
World	2,749,362,376	48.59	2,909,456,112	51.41
WHO region				
African region	22,676,140	3.68	593,557,990	96.32
Eastern Mediterranean region	8,221,320	1.76	458,884,199	98.24
European region	572,964,392	75.48	186,119,293	24.52
Region of the Americas	596,683,633	76.73	180,998,282	23.27
South-East Asia region	116,665,629	8.02	1,338,217,118	91.98
Western Pacific region	1,432,151,262	90.60	148,518,994	9.40
World Bank income classification				
High income	877,755,162	87.43	126,156,254	12.57
Upper middle income	1,658,636,497	77.72	475,526,821	22.28
Lower middle income	190,294,577	9.02	1,920,295,084	90.98
Low income	22,676,140	5.53	387,286,693	94.47

It is even more interesting to look at what proportion of the population was surveyed by region and income group. The most complete vaping survey coverage—over 90%—was in the Western Pacific region, driven by Australia, China, Japan, Malaysia, New Zealand, the Philippines and Taiwan. In Europe and the Americas, more than three-quarters of the population was surveyed. The rest of the world remains almost unknown. Only 8% of people in Asia were surveyed, 3.6% in Africa, and less than 2% in the Eastern Mediterranean.

As might be expected, this is closely related to the economic status of countries. In high-income countries, more than 87% of citizens were surveyed. In upper middle-income countries, the figure was not much lower at 77%. There is a huge gap between these and lower income countries, with only 9% of citizens surveyed in the latter. In the lowest income countries, less than 6% of citizens were surveyed.

### Estimation for countries without vaping survey data

Based on the available information from the surveys, we calculated the average prevalence of vaping for each WHO region, World Bank income classification group and the legal status of the sale of e-cigarettes in each country. Unfortunately, as we expected, some groups are very poorly represented (see Table 2). Low-income countries are only represented in the survey data by Uganda. Uganda is also the only data point for the African region. Similarly, we have only one data point from the South-East Asia region with Bangladesh, and the Eastern Mediterranean region with the United Arab Emirates.

**Table 2:** Average prevalence of vaping by factors used in the estimation

	Number of surveys	Prevalence of vaping use (%)
World Bank income classification		
High income	35	1.90
Upper middle income	11	1.21
Lower middle income	2	0.50
Low income	1	0.50
WHO region		
South-East Asia region	1	0.20
African region	1	0.50
Western Pacific region	8	1.34
European region	32	1.70
Region of the Americas	6	1.72
Eastern Mediterranean region	1	5.00
Legal status of e-cigarettes		
Banned	6	0.93
Allowed	40	1.75
No specific law	3	1.80

**Fig. 3:**
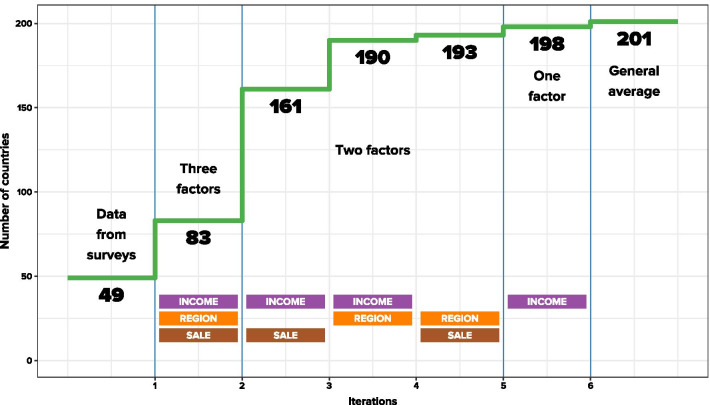
Information growth at subsequent levels of the estimation process

These three factors gave us four income groups, six regions and three sales statuses, which allowed us to separate 72 subgroups. For each of the groups the average prevalence of vaping was calculated. These 72 values were used as substitutes for the prevalence figures in the countries belonging to the group. Of course, not all subgroups were represented. For the first subdivision (1—in Fig. 3)—which was the most detailed, three-factor analysis—we had information for only 13 subgroups, which allowed us to calculate estimates for 83 countries.

For the other countries, we used a two-factor breakdown covering all pairs of these three factors. A second (2—in Fig. 3) split was made on the basis of income groups and sales status, which gave us eight information cells covering 161 countries, a third (3—in Fig. 3) was made on the basis of income groups and regions with 10 information cells covering 142 countries and a fourth (4—in Fig. 3) was made on the basis of regions and sales status, with nine information cells covering 102 countries. The last (5—in Fig. 3) subdivision was based only on one income-group factor.

The results of the calculations have been placed successively in the blanks remaining after the previous step. This means that the countries remaining without an estimated value after the first step have been assigned the values generated in the second step. In the third step we filled in the missing values remaining after the second step and in the fourth step filled in the missing values remaining after the third step. All remaining gaps were filled with the fifth step.

We started with 49 known countries. The first step increased this number to 83, the next to 161, the next to 190, the fourth one gave only three more countries (increasing the number of countries to 193), and the fifth to 198. There were still three countries left. We attributed the average value obtained from all known countries to these countries. Figure 3 shows detailed information of this process.

The number of vapers was calculated by taking as the denominator the total adult population (over 15 years old) obtained from the UN database for 2018 [2].

### Adjustment of the estimate for market value changes between 2018 and 2020

**Fig. 4:**
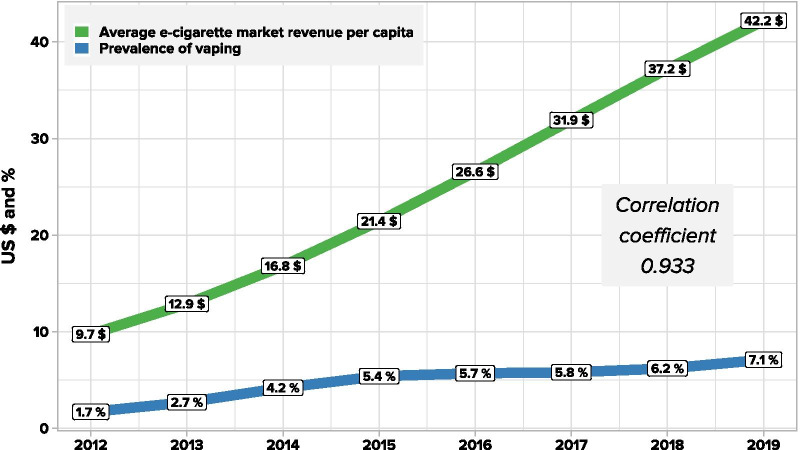
Correlation between average e-cigarette market revenue per capita and prevalence of vaping in the UK

Given the time lapse between the period when most of the surveys were undertaken and 2020, it is necessary to estimate subsequent growth. The only indicators available are estimates of market growth i.e. the value of sales. The question is, what is the relationship between e-cigarette market growth and changes in vaping prevalence? We have information on both variables for the UK for the period 2011 to 2019. We have used market-average e-cigarette market revenues per capita to avoid distortions related to population changes. As can be seen in Fig. 4, both trends were characterised by steady growth. The correlation between both series was very strong, with a Pearson correlation coefficient of 0.933. Based on this analysis, changes in e-cigarette market revenue are consistent with changes in vaping prevalence (with a correlation coefficient of 0.933). We assumed that this relationship is similar globally.

### Global estimation of vaping—2018

The number of vapers in the 49 countries from which the survey data are derived is 40.3 million. Using our estimation procedures we estimate that 17.8 million vapers live in the remaining 152 countries. This gives a total of 58.1 million vapers worldwide in 2018.

More than half of them live in high income countries. We estimate that 2.1 million vapers live in low income countries, 7.8 million in lower middle income countries, 19 million in upper middle income countries and 29.3 million in high income countries.

4.1 million vapers live in the African region, 4.2 million in the Eastern Mediterranean region, 4.6 million in the South-East Asia region, 11.2 million in the Western Pacific region, 15.3 million in the European region and 18.7 million in the region of the Americas.

38.6 million vapers live in countries where sale of e-cigarettes is allowed, 9.5 million in countries where sale of e-cigarettes is banned, and 10 million in countries where there is no specific law (see Table 3).

**Table 3:** Estimated number of vapers by income groups for 2018

	Number of countries	Number of vapers
World	201	58,107,606
Countries with data from surveys	49	40,334,650
Countries with estimated data	152	17,772,956
World Bank income classification		
Low income	31	2,115,585
Lower middle income	47	7,760,169
Upper middle income	54	18,959,299
High income	54	29,269,384
Unknown	1	3,169
WHO regions		
African region	47	4,107,918
Eastern Mediterranean region	21	4,199,293
South-East Asia region	11	4,554,551
Western Pacific region	23	11,150,297
European region	50	15,345,345
Region of the Americas	33	18,741,095
Unknown	2	9,107
Legal status of e-cigarettes		
Allowed	72	38,648,243
Banned	36	9,458,630
No specific law	79	10,000,733

### Projection of the estimated global number of vapers—2020

The projection for the year 2020 was made on the basis of information from the *E-Cigarettes Worldwide Statista Market Forecast* [18]. The global number of vapers estimate was fitted to revenue data at the 2018 time point (meaning revenue data from 2018 was directly paired with estimated number of vapers). The market trend was zeroed (calibrated) at 2018. The proportions series was adjusted with the above-mentioned coefficient of correlation between average e-cigarette market revenue and number of vapers (see Table 4).

**Table 4:** Average e-cigarette market revenue trend in percent centred at 2018

						Point zero		Target point			
2012	2013	2014	2015	2016	2017	2018	2019	2020	2021	2022	2023
37%	44%	52%	62%	74%	87%	100%	110%	117%	129%	139%	148%

**Fig. 5:**
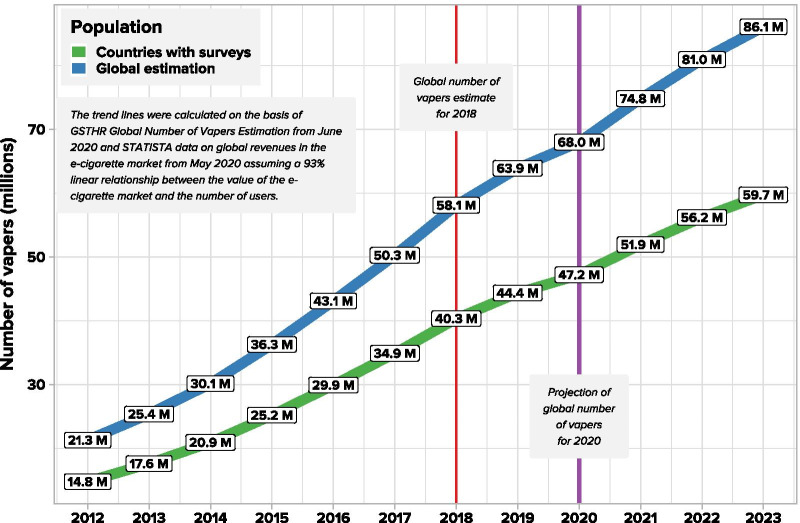
Estimated trends in the worldwide number of vapers

Multiplying subsequent proportions by the estimated global number of vapers, we have given projections of this number for other years. For the year 2020, the projection is 68 million vapers globally (see Fig. 5).

## Discussion

We estimated that there were approximately 68 million users of e-cigarettes worldwide in 2020. This estimate should be treated with caution, as it is based on extrapolation of data from 49 countries; data were missing for 152 countries and territories (over half of the world population). We use a method commonly used in global epidemiological studies that assumes similarity between countries with shared characteristics. The robustness of this assumption needs to be tested, and it must be determined whether the method is suitable for estimating consumer behaviour where there are many factors at play including social, economic, cultural, commercial and legal considerations. The small number of data points for some world regions and World Bank classifications is a further weakness.

There is also wide variation in survey definitions of vaping. It is obvious from the data collected and assessed in our database that the available information is often of poor quality—survey samples are sometimes insufficiently large to provide sufficient precision to measure such small sections of the population. The sampling method of many studies is not probabilistic, which makes it impossible to generalise the measured values correctly per population. It is well known that monitoring risk behaviours in relatively small or hidden populations is not straightforward.

These limitations indicate a surprising deficiency in the national and hence global monitoring of vaping. Despite the WHO's call for countries to implement the MPOWER strategy—recall that the first letter in the strategy's name refers to monitoring—global monitoring of tobacco use is weak. Only 38% of the global population is covered by surveys of tobacco use [21], and only 1 in 3 countries monitors tobacco use by repeating nationally representative youth and adult surveys at least once every 5 years [22]. Moreover, despite the fact there is considerable global and international debate about e-cigarettes, most countries are taking no steps to monitor use at a national level. Evidence based policymaking is very difficult in the absence of evidence. Large sums of money have been invested by international philanthropic organisations, including Bloomberg Philanthropies, to oppose tobacco harm reduction using e-cigarettes and other SNP. It is perhaps ironic that these initiatives do not include any impact measure. Efforts to improve the coverage and accuracy of information about vaping should be encouraged.

## Conclusions

Our best estimate of 68 million vapers indicates considerable uptake given that: the products have only been available on most markets for 10 years or less; there is either no support or there is opposition to vaping in many countries; and in countries which regulate e-cigarettes there may be strict controls over advertising and promotion. With this work, we can compare the prevalence of SNP use and smoking and determine the extent of tobacco harm reduction. Given the global scale of tobacco smoking (at 1.1 billion people globally) progress in adoption of alternative products is slow. The number of people using e-cigarettes is still a small fraction of the number of people who smoke.

Historically, changes in nicotine consumption can take many decades. For example the last major disruptive innovation for tobacco was the invention of the cigarette rolling machine in the 1880s, but it took a further 60 years or so for the machine rolled cigarette to replace most other forms of tobacco use in richer countries. Given the public and individual health imperative to either quit or switch from combustible to non-combustible nicotine products, there is an urgent need to scale up tobacco harm reduction.

## Supplementary Information

**Additional file 1.** List of surveys on nicotine vaping.

## Data Availability

Source datasets supporting the conclusions in this article are available in various repositories. See the Additional file 1: List of surveys on nicotine vaping for detailed sources and references. The aggregated dataset is available at https://gsthr.org/countries.

## Abbreviations

NRT: Nicotine replacement therapy.
SNP: Safer nicotine products.
GSTHR: Global State of Tobacco Harm Reduction

## Footnotes

1 A new special Eurobarometer 506 was published in February 2021 [6]. Comparing our projection to 2020 (GNV) and the Eurobarometer data from 2020 (EB), we have 16 countries where the prevalence of vaping are greater in EB than GNV; 12 countries where prevalence are less in EB than GNV. The differences are small. On average, GNV is underestimated relative to EB by about 0.29 percentage points.
