# Supplementary material for: Estimation of the global number of e-cigarette users in 2020
Source: Harm Reduct J. 2021 Oct 23;18:109. doi: 10.1186/s12954-021-00556-7 (PMC8541798; doi:10.1186/s12954-021-00556-7)
Supplement: Supplementary file 1 — Additional file 1. List of surveys on nicotine vaping. [file 12954_2021_556_MOESM1_ESM.docx]

Supplementary table to the “Estimation of the global number of nicotine vapers in 2020”

Surveys on nicotine vaping

| Country | Year | References |
| --- | --- | --- |
| Australia | 2016 | Australian Institute of Health and Welfare 2017. National Drug Strategy Household Survey 2016: detailed findings. Drug Statistics series no. 31. Cat. no. PHE 214. Canberra: AIHW |
| Austria | 2017-2018 | European Commission (2017). Special Eurobarometer 458: Attitudes of Europeans towards tobacco and electronic cigarettes [1] |
| Bangladesh | 2017 | Global Adult Tobacco Survey, 2017 [2] |
| Belgium | 2017-2018 | European Commission (2017). Special Eurobarometer 458: Attitudes of Europeans towards tobacco and electronic cigarettes |
| Brazil | 2015 | Bertoni, N., Szklo, A., Boni, R. D., Coutinho, C., Vasconcellos, M., Nascimento Silva, P., de Almeida, L. M., & Bastos, F. I. (2019). Electronic cigarettes and narghile users in Brazil: Do they differ from cigarettes smokers? Addictive Behaviors, 98, 106007. https://doi.org/10.1016/j.addbeh.2019.05.031 |
| Bulgaria | 2017 | European Commission. (2017). Special Eurobarometer 458: Attitudes of Europeans towards tobacco and electronic cigarettes |
| Canada | 2013 | Reid J L. Rynard V L. Czoli C D. Hammond D.(2015). Who is using e-cigarette in Canada? Nationally representative data on the prevalence of e-cigarette use among Canadians. Preventative medicine. 81. 180-183. |
| China | 2015 | Feng, G., Nan, Y. and Jiang, Y., 2018, January. Prevalence of e-cigarette in China: preliminary findings from two surveys. In TOBACCO INDUCED DISEASES (Vol. 16, pp. 102-102). |
| Colombia | 2015 | no-data |
| Costa Rica | 2015 | Global Adult Tobacco Survey, 2015. |
| Croatia | 2017 | European Commission. (2017). Special Eurobarometer 458: Attitudes of Europeans towards tobacco and electronic cigarettes |
| Cyprus | 2017-2018 | European Commission (2017). Special Eurobarometer 458: Attitudes of Europeans towards tobacco and electronic cigarettes |
| Czech Republic | 2017-2018 | European Commission (2017). Special Eurobarometer 458: Attitudes of Europeans towards tobacco and electronic cigarettes |
| Denmark | 2017-2018 | European Commission (2017). Special Eurobarometer 458: Attitudes of Europeans towards tobacco and electronic cigarettes |
| Estonia | 2017-2018 | European Commission (2017). Special Eurobarometer 458: Attitudes of Europeans towards tobacco and electronic cigarettes |
| Finland | 2017-2018 | European Commission (2017). Special Eurobarometer 458: Attitudes of Europeans towards tobacco and electronic cigarettes |
| France | 2017-2018 | European Commission (2017). Special Eurobarometer 458: Attitudes of Europeans towards tobacco and electronic cigarettes |
| Germany | 2017-2018 | European Commission (2017). Special Eurobarometer 458: Attitudes of Europeans towards tobacco and electronic cigarettes |
| Greece | 2017 | 1. Farsalinos K. Siakas G. Poulas K. Voudris V. Merakou K.and Barbouni A. (2018). Electronic cigarette use in Greece: an analysis of a representative population sample in Attica prefecture. Harm Reduction Journal. 15:20 Available at: https://harmreductionjournal.biomedcentral.com/articles/10.1186/s12954-018-0229-7. 2. European Commission (2017). Special Eurobarometer 458: Attitudes of Europeans towards tobacco and electronic cigarettes |
| Hong Kong | 2018 | Thematic Household Survey Report No. 64 issued in April 2018. Available at: https://www.taco.gov.hk/t/english/infostation/infostation_ec.html |
| Hungary | 2017-2018 | European Commission (2017). Special Eurobarometer 458: Attitudes of Europeans towards tobacco and electronic cigarettes |
| Iceland | 2018 | Iceland direcorate of Health Newsletter, reported in Iceland Review. Available at:http://icelandreview.com/news/2018/05/03/vaping-linked-decrease-cigarette-smoking |
| Ireland | 2019 | Healthy Ireland Survey documents. https://www.gov.ie/en/collection/231c02-healthy-ireland-survey-wave/ (2019). |
| Italy | 2017 | European Commission. (2017). Special Eurobarometer 458: Attitudes of Europeans towards tobacco and electronic cigarettes |
| Japan | 2017 | Tabuchi, T. et al (2017) Heat-not-burn tobacco product use in Japan: its prevalence, predictors and perceived symptoms from exposure to secondhand heat-not-burn tobacco aerosol. Tobacco Control. https://tobaccocontrol.bmj.com/content/early/2017/12/15/tobaccocontrol-2017-053947?papetoc=#DC1 |
| Kazakhstan | 2014 | WHO (2014) Global Adult Tobacco Survey (GATS) .The Republic of Kazakhstan, 2014. Country Report. |
| Latvia | 2017-2018 | European Commission (2017). Special Eurobarometer 458: Attitudes of Europeans towards tobacco and electronic cigarettes |
| Lithuania | 2017-2018 | European Commission (2017). Special Eurobarometer 458: Attitudes of Europeans towards tobacco and electronic cigarettes |
| Luxembourg | 2017-2018 | European Commission (2017). Special Eurobarometer 458: Attitudes of Europeans towards tobacco and electronic cigarettes |
| Malaysia | 2016 | Ab Rahman, J. et al. (2019) "The Prevalence of E-Cigarette Use Among Adults in Malaysia: Findings From the 2016 National E-Cigarette Survey", Asia Pacific Journal of Public Health. doi: 10.1177/1010539519834735. |
| Malta | 2017-2018 | European Commission (2017). Special Eurobarometer 458: Attitudes of Europeans towards tobacco and electronic cigarettes |
| Mexico | 2017 | Encuesta Nacional de Consumo de Drogas, Alcohol y Tabaco, ENCODAT 2016-2017. Available at:https://drive.google.com/file/d/1Iktptvdu2nsrSpMBMT4FdqBIk8gikz7q/view |
| Netherlands | 2017-2018 | European Commission (2017). Special Eurobarometer 458: Attitudes of Europeans towards tobacco and electronic cigarettes |
| New Zealand | 2016 | Oakly et al. 2019.Prevalence of e-cigarette use from a nationally representative sample in New Zealand. Addictive Behaviours. Volume 98, November 2019, 106024. Available at: https://www.sciencedirect.com/science/article/abs/pii/S0306460318314102?via%3Dihub |
| Philippines | 2015 | Health Survey Northern Ireland. 2017/2018. Available at:https://www.health-ni.gov.uk/publications/health-survey-northern-ireland-first-results-201718 |
| Poland | 2017 | European Commission (2017). Special Eurobarometer 458: Attitudes of Europeans towards tobacco and electronic cigarettes |
| Portugal | 2017-2018 | European Commission (2017). Special Eurobarometer 458: Attitudes of Europeans towards tobacco and electronic cigarettes |
| Romania | 2017-2018 | European Commission (2017). Special Eurobarometer 458: Attitudes of Europeans towards tobacco and electronic cigarettes |
| Russian Federation | 2016 | Global Adult Tobacco Survey, 2016. |
| Serbia | 2017 | Kilibarda, B. et al, (2018) E-cigarette use in Serbia: Prevalence, reasons for trying and perceptions. Addictive Behaviors. |
| Slovakia | 2017-2018 | European Commission. (2017). Special Eurobarometer 458: Attitudes of Europeans towards tobacco and electronic cigarettes |
| Slovenia | 2017-2018 | European Commission (2017). Special Eurobarometer 458: Attitudes of Europeans towards tobacco and electronic cigarettes |
| Spain | 2017-2018 | European Commission (2017). Special Eurobarometer 458: Attitudes of Europeans towards tobacco and electronic cigarettes |
| Sweden | 2018 | Public Health Agency. National Public Health Survey (tobacco/e-cigarette) 2018. |
| Taiwan | 2014 | Chen, Y.L., et al. (2018) E-Cigarette use in a country with prevalent tobacco smoking: A population based study in Taiwan. Journal of Epidemiology, 2018. doi: 10.2188/jea.JE20170300. [Epub ahead of print]. |
| Uganda | no data | no data |
| United Arab Emirates | no data | no data |
| United Kingdom | 2019 | Action on Smoking and Health (ASH). (2019). Use of e-cigarettes (vaporisers) among adults in Great Britain. 2019 (September), 1–15. |
| United States | 2020 | Al Rifai M, et al. Abstract 1209-110. Presented at: American College of Cardiology Scientific Session; March 28-30, 2020 (virtual meeting). |

1. European Commission. Special Eurobarometer 458: Attitudes of Europeans towards tobacco and electronic cigarettes [Internet]. 2017. Available from: https://data.europa.eu/euodp/en/data/dataset/S2146_87_1_458_ENG

2. WHO. Global Adult Tobacco Survey (GATS) [Internet]. World Health Organization; 2017. Available from: http://www.who.int/tobacco/surveillance/survey/gats/en/
